# Supplementary material for: Conditional chemoconnectomics (cCCTomics) as a strategy for efficient and conditional targeting of chemical transmission
Source: eLife. 2024 Apr 30;12:RP91927. doi: 10.7554/eLife.91927 (PMC11060718; doi:10.7554/eLife.91927)
Supplement: Figure 3—source data 3. [file elife-91927-fig3-data3.zip › Figure 3 source data3 part 1.zip!/Figure 3-Source data 3-part1/1063f3-1-CRISPResso_Report_kboyr13q.zip!/CRISPResso_on_1063f3_3/1c.Alignment_barplot.pdf]

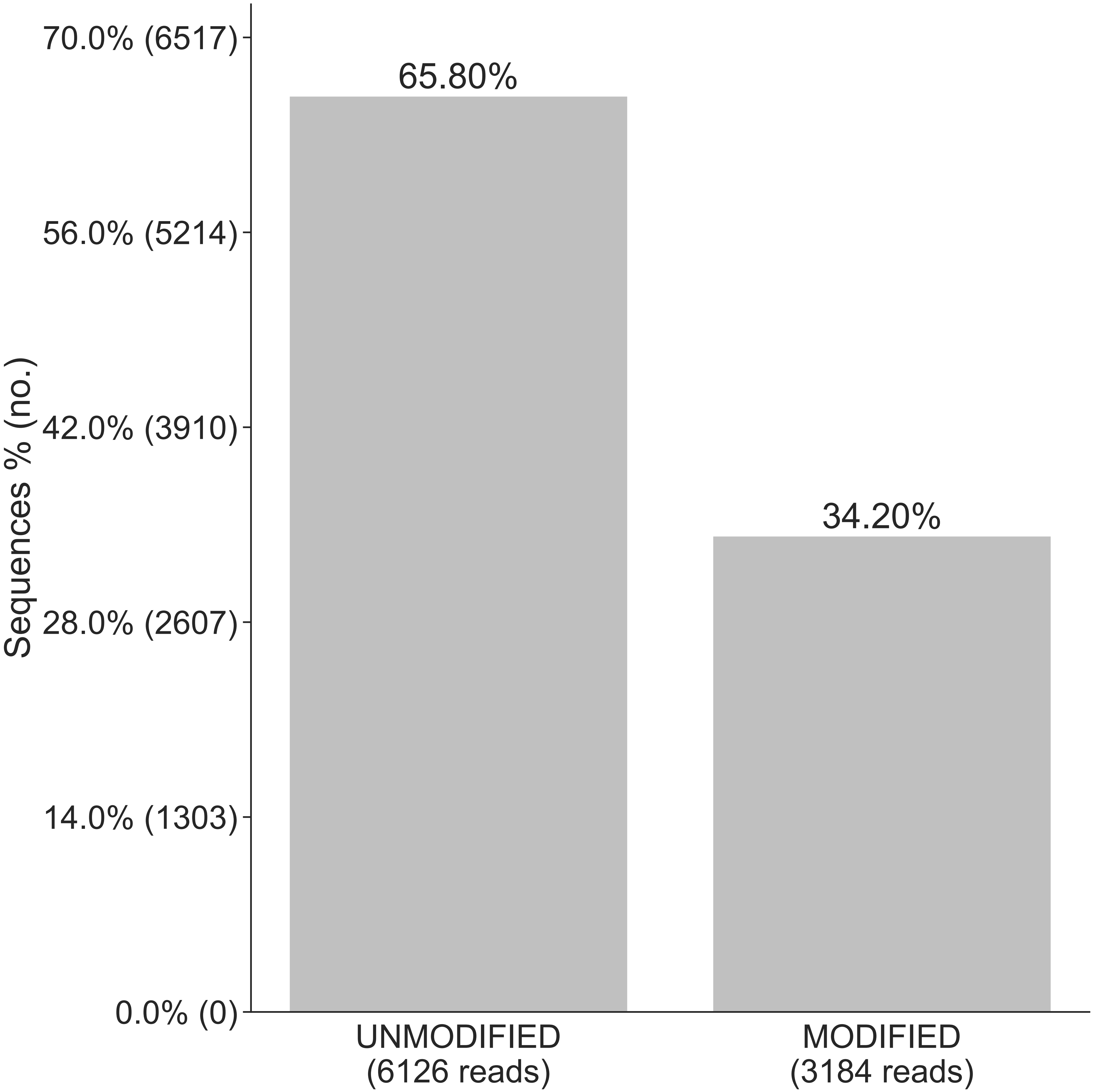

70.0% (6517)
56.0% (5214)
42.0% (3910)
28.0% (2607)
14.0% (1303)
0.0% (0)
Sequences % (no.)
65.80%
34.20%
UNMODIFIED
(6126 reads)
MODIFIED
(3184 reads)
